# Improving maize yield in newly reclaimed soils: effects of irrigation, mulching, and foliar treatments

**DOI:** 10.1186/s12870-025-06637-0

**Published:** 2025-05-14

**Authors:** Ahmed M. Abdelghany, A. S. Farouk, E. Sh. Alwakel, Mohamed Ebaid, Mahmoud Naser, Sobhi F. Lamlom, A. A. Shehab

**Affiliations:** 1 Crop Science Department, Faculty of Agriculture, Damanhour University, Damanhour, Egypt; 2 Agronomy Department, Faculty of Agriculture, Al-Azhar University, Cairo, Egypt; 3 Plant Production Department, Arid Lands Cultivation Research Institute (ALCRI), City of Scientific Research and Technological Applications (SRTA-City), New Borg El-Arab City, Alexandria, 21934 Egypt; 4 Plant Production Department, Faculty of Agriculture Saba Basha, Alexandria University, Alexandria, 21531 Egypt

**Keywords:** Maize, Drip irrigation, Mulching, Potassium bicarbonate, Multivariate analysis

## Abstract

Improving maize yield in newly reclaimed soils presents major challenges. This study analyzed the impact of various irrigation methods (drip, sprinkler, and surface), foliar applications (potassium bicarbonate (PoB), methanol, and water control), and mulching techniques (with and without rice paddy straw) on the growth, physiology, productivity, and water use efficiency (WUE) of maize in newly reclaimed soil over the two seasons of 2022 and 2023. The study employed a split-split plot design arranged in randomized complete blocks with three replications. The integration of drip irrigation, PoB foliar spray, and rice paddy straw mulching yielded superior results, improving grain yield (GY,10.0-13.08 t/ha) and biological yield (BY, 51.37–59.53 t/ha) in comparison to control treatments. The findings were validated through various multivariate analyses, such as principal component analysis (PCA), structural equation modeling (SEM), hierarchical clustering heatmap, and radar plot visualization. The findings of PCA revealed that the initial two components accounted for 85.6% of the total variance, with growth and yield parameters exhibiting a strong correlation with the first principal component (76.8%). Also, SEM analysis revealed that BY (0.603), plant height (0.594), and net assimilation rate (0.589) exerted the most significant direct positive influences on GY. Drip irrigation demonstrated the highest WUE at 3.70 kg/m³, significantly surpassing sprinkler irrigation at 3.32 kg/m³ and surface irrigation at 3.03 kg/m³. Mulched plots showed better WUE (3.53 kg/m³) compared to non-mulched plots (3.17 kg/m³), with PoB application yielding the highest WUE (3.80 kg/m³). Seasonal variations were noted, with drip irrigation combined with mulch and PoB reaching the highest WUE (4.20 kg/m³) in the first season, whereas sprinkler irrigation with mulch and PoB demonstrated better performance (5.49 kg/m³) in the second season. The results highlight the opportunity to enhance maize yields in newly reclaimed soils, with the integration of drip irrigation, potassium bicarbonate foliar application, and rice straw mulching emerging as the most innovative practice for boosting crop performance and optimizing water use.

**Supplementary Information:**

The online version contains supplementary material available at 10.1186/s12870-025-06637-0.

## Clinical trial number

Not applicable (NA).

**Supplementary Information:**

The online version contains supplementary material available at 10.1186/s12870-025-06637-0.

## Introduction

The production of maize (*Zea mays* L.) is essential for food security and economic stability in several places, especially in emerging nations. Maize, one of the most extensively farmed crops worldwide, is an essential food source and raw material for several industries. Its versatility under various environmental situations renders it crucial for guaranteeing food security [1]. Maize productivity is frequently impeded by adverse conditions, particularly in newly reclaimed sandy soils, which exhibit low water retention, elevated evaporation rates, and nutrient deficiencies [2, 3]. These challenges underscore the necessity for innovative agricultural strategies to enhance crop performance. Research indicates that effective management techniques can substantially improve maize productivity and its resilience to environmental stressors [4].

Farming on newly reclaimed sandy soils presents specific challenges. These soils are typically susceptible to erosion and water loss, exhibit low organic matter content, and possess limited nutrient availability [5]. In these regions, farmers must implement efficient irrigation techniques and soil management practices to mitigate the soil’s elevated porosity and inadequate water retention ability [6]. Earlier research indicated that maize yields on sandy soils may be significantly inferior than those on more fertile soils if the land is inadequately maintained [7]. In this concern, irrigation significantly affects crop yield in arid and semi-arid regions. The selection of irrigation technique significantly influences water-use efficiency and agricultural output [8]. Conventional surface irrigation methods frequently result in water loss due to evaporation and runoff [9]. In contrast, contemporary techniques like drip and sprinkler irrigation facilitate precise water application, enhancing water efficiency [10–12]. Studies demonstrate that drip irrigation conserves water and enhances nutrient absorption by delivering water directly to the root zone, particularly beneficial in sandy soils with low moisture retention [13].

Mulching is a beneficial agronomic practice that enhances soil moisture retention, minimizes evaporation, suppresses weed growth, and improves soil health [14, 15]. Rice straw used as mulch significantly enhances soil structure and microbial activity, resulting in improved nutrient availability for crops [16]. Rice straw mulch serves as a protective layer on the soil surface, reducing erosion from wind and water and regulating soil temperature variations [17]. Furthermore, it was reported that rice straw mulch improves soil physical properties by augmenting soil organic carbon levels and enhancing moisture retention [18]. In addition, previous studies indicated that the application of rice straw mulch results in increases in soil moisture content between 3% and 9% [19]. This effect is vital for sandy soils, as sustaining appropriate moisture levels is critical for plant growth. A study on sunflower found that applying rice straw mulch increased yield by 23%, primarily due to enhanced root development [20]. The study also revealed that rice straw mulch improved soil water content by 3–9% compared to the no-mulch treatment.

Foliar applications serve as a method for enhancing nutrient availability during essential growth phases. Research indicates that the application of methanol significantly influences crop growth and physiological parameters [21]. For example, methanol sprays (20–30% concentration) were found to enhance photosynthetic efficiency by increasing CO2 fixation and reducing photorespiration, leading to improved leaf area index and chlorophyll content in *Phaseolus vulgaris* L [22]. Studies indicated that methanol with a concentration of 10% maximized seed yield, while drought-stressed plants responded best to 30% methanol application, showing improved growth and yield parameters. In maize, foliar application timing proved crucial, with treatments during multiple growth stages (8–10 leaf, tasseling, and seed-filling) significantly affecting nutrient reserves (N, Mg, Zn, Mn, B) and subsequent generation seed yield, achieving optimal results when combined with other nutrients [23–25]. In Canola, methanol foliar spray enhanced growth and yield parameters under rainfed conditions, with 10% concentration showing optimal results in pod number, seed yield, and plant height. The beneficial effects are attributed to methanol’s metabolism to CO_2_ and water in plant tissues, which reduces photorespiration [26].

Potassium bicarbonate (PoB) has become an important foliar spray treatment in agriculture, specifically for improving crop yield and quality. Recent studies demonstrate that PoB application can enhance grain protein content by 8–12%, significantly contributing to the nutritional value of crops including maize [27], wheat [28], and faba bean [29]. Furthermore, it has been demonstrated to alleviate oxidative stress in wheat by decreasing markers such as hydrogen peroxide and malondialdehyde by more than 30% [25, 30]. Furthermore, it has been demonstrated to alleviate oxidative stress in wheat by decreasing markers such as hydrogen peroxide and malondialdehyde by more than 30% [30]. The reduction of oxidative stress is essential for sustaining plant health and enhancing resilience to environmental stressors [31]. The benefits are attributed to PoB’s function as a potassium source, crucial for numerous physiological processes in plants, such as enzyme activation and photosynthesis. Additionally, its use has been associated with increased enzymatic activity in antioxidant defense systems, thus enhancing overall plant vigor and productivity [32]. The application of PoB enhances crop yield and promotes sustainable agricultural practices by minimizing reliance on chemical fungicides, as it effectively manages fungal diseases without inducing phytotoxicity [33].

In this study, we investigated how different irrigation methods (surface, sprinkler, and drip) interact with mulching practices and foliar applications to affect maize performance in newly reclaimed sandy soils. We sought to determine which combination of management practices yield the optimal water use efficiency and productivity for maize cultivation in challenging soil conditions. Additionally, we examined the underlying mechanisms and relationships between these management practices and plant physiological responses. We hypothesized that drip irrigation would outperform other irrigation methods in terms of water use efficiency and yield enhancement, particularly when combined with mulching. We expected that rice straw mulch would significantly improve soil moisture retention and reduce temperature fluctuations, leading to enhanced plant growth parameters. Furthermore, we anticipated that potassium bicarbonate foliar application would provide superior benefits compared to methanol by improving plant physiological functions and stress resilience. Finally, we hypothesized that the integration of all three optimal management practices would demonstrate synergistic effects greater than the sum of their individual benefits.

Specifically, the objectives of this study were to evaluate the individual and combined effects of irrigation methods, mulching practices, and foliar applications on maize growth, physiology, and productivity in newly reclaimed sandy soil. We aimed to determine the most water-efficient management combination for sustainable maize production, assess the relationships between management practices and plant responses using multivariate analytical methods, and identify optimal integrated management strategies for improving maize productivity in resource-limited conditions.

## Materials and methods

### Experimental site and environmental conditions

**Fig. 1:**
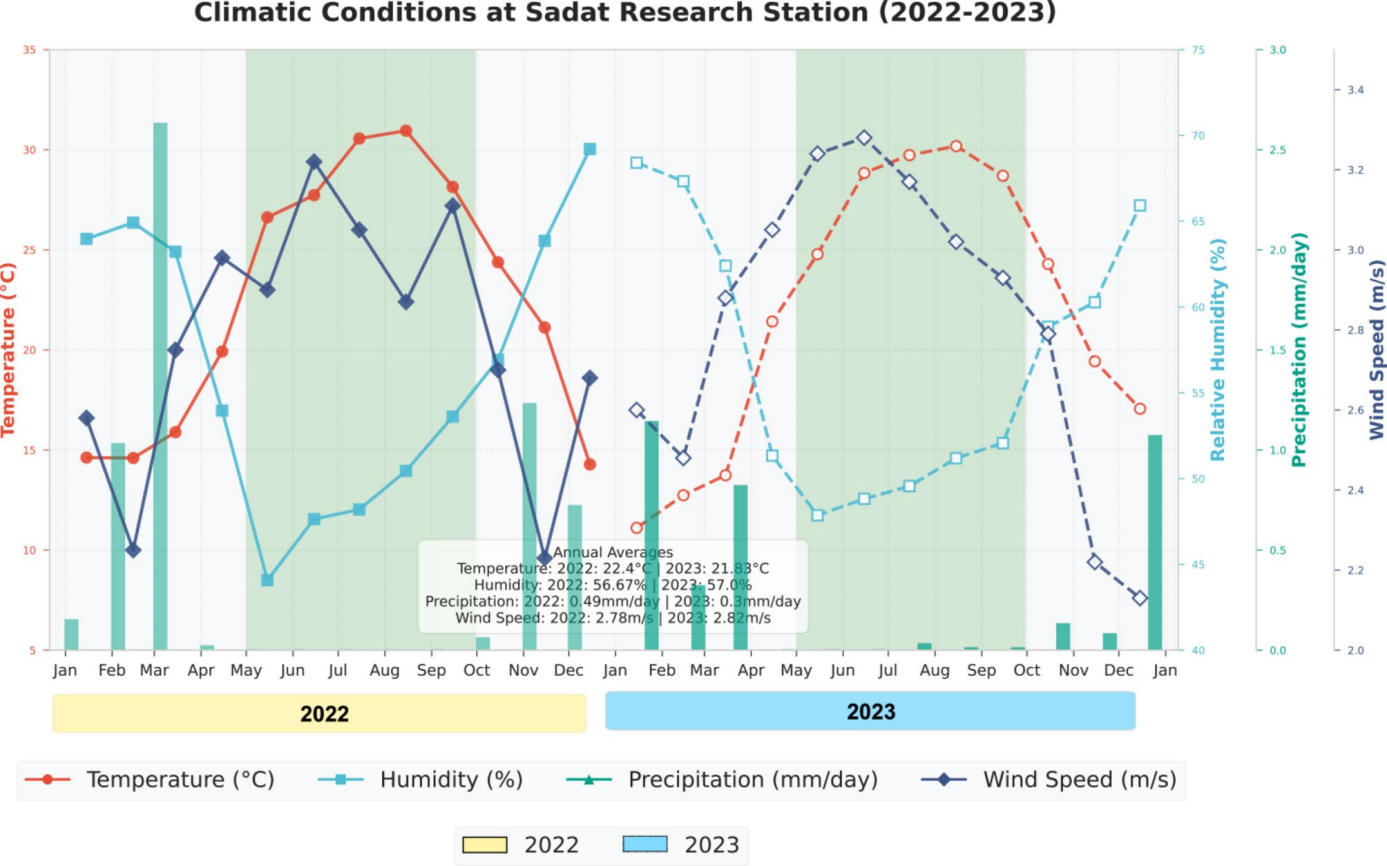
Climatic Conditions at Sadat Research Station (2022–2023): Monthly trends of temperature (°C), relative humidity (%), precipitation (mm/day), and wind speed (m/s) during 2022 and 2023 growing seasons. Green-shaded areas indicate maize growing periods (May-September). Data points represent monthly averages with solid lines for 2022 and dashed lines for 2023, highlighting seasonal patterns and inter-annual variability in meteorological parameters influencing maize cultivation in newly reclaimed sandy soil

The study was conducted at the Sadat Research Station of Al-Azhar University (30.5266′N, 30.3811′E, 21 m above sea level) in Sadat, Al Manoufiya Governorate, Egypt. The experimental site is characterized by an arid climate with minimal rainfall concentrated primarily in winter months (Fig. 1). During the study period (2022–2023), average annual precipitation was low (0.49 mm/day in 2022 and 0.30 mm/day in 2023), with most rainfall occurring between November and March. The area experiences hot summers and mild winters, with average annual temperatures of 22.4 °C in 2022 and 21.83 °C in 2023. Summer temperatures peaked in July-August (exceeding 30 °C), while winter temperatures dropped to their lowest in January-February (11–15 °C). Relative humidity averaged 56.67% and 57.00% in 2022 and 2023, respectively, with higher values during winter months. Average annual wind speed was consistent across both seasons at approximately 2.8 m/s. The experimental soil is classified as newly reclaimed sandy soil with low organic matter content (0.64% in 2022 and 0.63% in 2023). Soil texture analysis showed predominance of sand fractions (fine sand: 36.60-38.28%; coarse sand: 43.61–44.12%) with small proportions of silt (11.74–13.44%) and clay (4.24–6.75%). The soil had a low calcium carbonate content (0.60–0.62%) and a slightly alkaline pH (7.9-8.0) as shown in Table 1.

The soil analysis revealed moderate to low nutrient availability, typical of newly reclaimed sandy soils in arid regions as confirmed by the data shown in Table 1. These soil samples were collected using a soil auger, air-dried, ground, and passed through a 2 mm sieve according to standard procedures [34]. Physical properties (soil texture, fine sand, coarse sand, silt, and clay percentages) were determined using the hydrometer method [35]. Chemical analyses were determined following Black et al. [34]. Nitrogen content was relatively low (49 ppm in 2022 and 45 ppm in 2023), which is consistent with the low organic matter content. Phosphorus availability was also low (8.2–9.4 ppm), while potassium levels were moderate (207–218 ppm). The soil had low electrical conductivity (1.1 mmhos/cm in both seasons), indicating minimal salinity issues. The cation exchange was dominated by sodium (Na⁺: 5.25–5.49) and calcium (Ca²⁺: 2.5–2.6), with lower concentrations of magnesium (Mg²⁺: 2.1–2.3) and potassium (K⁺: 0.61–0.62). The bicarbonate (HCO₃⁻) concentration was 2.3–2.6, while chloride (Cl⁻) and sulfate (SO₄²⁻) levels were 8.4–8.5 and 1.2–1.4, respectively. These nutrient levels underscore the challenges of crop production in newly reclaimed soils and highlight the importance of exploring optimized management practices to enhance maize productivity in such environments.

As for the cropping history of the experimental site, these plots were part of a regular crop rotation system in the newly reclaimed soil. Prior to the maize experiment, the plots were cultivated with faba bean during the winter season, and following the maize experiment, Egyptian clover was planted. This rotation system is typical for newly reclaimed soils in Egypt, where leguminous crops like faba bean and Egyptian clover are incorporated to improve soil fertility through nitrogen fixation, particularly important in sandy soils with inherently low nutrient content. Furthermore, experimental design was established with careful consideration of field variations. Based on preliminary soil analysis and topographical assessment, a unidirectional variation was identified across the experimental area. Consequently, the experimental plots were arranged vertically along this direction of variation to account for potential soil heterogeneity.

**Table 1:** Physical and chemical properties of the upper 50 cm of the experimental soil sites

**Season**	**Fine sand %**	**Coarse sand %**			**Silt %**		**Clay %**		**CaCo**_**3**_ **%**	**O.M %**		**Soil texture**
2022	36.60	44.12			13.44		4.24		0.6	0.64		Sandy
2023	38.28	43.61			11.74		6.75		0.62	0.63		
**Season**	**EC** **(mmhos / cm)**	**pH**	**HCO**_**3**_	**Cl** ^**−**^	**So**_**4**_^**−−**^	**Ca** ^**++**^	**Mg** ^**++**^	**Na** ^**+**^	**K** ^**+**^	**N (ppm)**	**P** **(ppm)**	**K (ppm)**
2022	1.1	7.9	2.6	8.5	1.2	2.5	2.1	5.49	0.61	49	8.2	218
2023	1.1	8	2.3	8.4	1.4	2.6	2.3	5.25	0.62	45	9.4	207

### Experimental design, treatments

#### Experimental design

The experiment utilized a split-split plot design arranged in a randomized complete blocks with three replications. The primary plots were designated for three irrigation techniques (drip, sprinkler, and surface irrigation), sub-plots for mulching treatments (with and without rice straw mulch), and sub-sub plots for foliar spray applications (water (CK), methanol solution, and potassium bicarbonate (PoB)) as illustrated in supplementary Fig. S1. The area of the experimental unit was 21 m² (3 × 7 m), with a total experimental area of 1134 m², inclusive of boundaries and corridors.

#### Treatments details

The experimental irrigation systems comprised drip, sprinkler, and furrow irrigation techniques. The drip irrigation system incorporated pressure-compensating drip lines (GR model) with an emission rate of 4 L h⁻¹ and an emitter spacing of 0.5 m. Impact sprinklers were utilized for sprinkler irrigation, installed on 1.2-meter risers, functioning at an operating pressure of 250 kPa (2.5 bars). Surface irrigation was implemented with level furrows as the traditional irrigation technique. To ensure experimental consistency, all irrigation systems were calibrated to provide a seasonal irrigation volume of 3000 m³ ha⁻¹. Mulch treatments involved the application of rice straw at a rate of 5 Mg ha⁻¹, creating a consistent layer of roughly 50 mm in thickness. Mulch was applied after crop emergence and before the first watering event. Control treatments were conducted without the application of mulch. The three foliar treatments included simple water spray (control), a 20% aqueous methanol solution, and a PoB solution (0.07 g/L). All foliar sprays were conducted using a 20 L capacity backpack sprayer, fitted with a flat-fan nozzle working at a pressure of 3 bars. To reduce quick evaporation, treatments were performed in the early morning (before 9:00 AM) at four development stages: 15, 30, 45, and 60 days after sowing (DAS). A consistent spray volume of 600 L/ha was kept across all treatments.

The maize hybrid utilized in this investigation was the single-cross HYTECH2036, a white single-cross variety, sourced from Misr Hytech Seed International Company. It was planted on April 15th in both seasons. Seeds were manually sown at 25 cm intervals inside rows spaced 70 cm apart, resulting in a plant density of around 50,000 plants per hectare. Prior to planting, the experimental field was prepared through double plowing to a depth of 30 cm, followed by harrowing and leveling. Weeds were managed through pre-emergence application of Stomp^®^ (pendimethalin) at 1.7 L/ha, followed by two hand weeding operations at 21 and 45 days after sowing (DAS). Pest management included application of Lambda^®^ (lambda-cyhalothrin) at 400 mL/ha at 30 and 45 DAS to control stem borer (*Sesamia cretica*) and armyworm (*Spodoptera littoralis*). Fertilization was conducted in accordance with local guidelines: 285 kg N/ha using ammonium nitrate (33.5% N), 200 kg P₂O₅/ha utilizing calcium superphosphate (15.5% P₂O₅), and 120 kg K₂O/ha employing potassium sulfate (48% K₂O). Phosphorus was included during soil preparation, whereas nitrogen and potassium were administered in three equal applications at 21, 35, and 50 DAS. Rice straw mulch, where applicable, was applied at 5 t/ha after crop emergence. Foliar treatments were applied three times at 30, 45, and 60 DAS using a backpack sprayer. Irrigation scheduling was based on soil moisture sensors, maintaining 70–80% of field capacity. Harvesting was performed manually when grain moisture content reached approximately 15–18% (110–115 DAS). All treatments adhered to standardized agronomic techniques in accordance with regional standard requirements.

### Data collection and measurements

#### Growth parameters

Plant height (PH) was quantified from the ground level to the base of the tassel. The evaluation of net assimilation rate (NAR) and crop growth rate (CGR) was conducted using the below formulae [36]:

$$\:\text{NAR}\:=\frac{\:(\text{W2}\:-\:\text{W1})/(\text{T2}\:-\:\text{T1})\:\times\:\:(\text{lnA2}\:-\:\text{lnA1})}{(\text{A2}\:-\:\text{A1})}$$

$$\:CGR\:=\:(W2\:-\:W1)/\:(T2\:-\:T1)\:\times\:\:1/GA$$

Where: W1, W2 = dry weights at times T1 and T2 A1, A2 = leaf areas at times T1 and T2 GA = ground area Measurements were taken at 30 and 60 DAS.

#### Physiological measurements comment 9

The leaf area index (LAI) was measured with a LAI-2200 C Plant Canopy Analyzer (LI-COR Inc., USA) between 10:00 and 12:00 h under consistent diffused light conditions, as outlined by Welles and Norman [37]. Leaf water content (LWC) was determined following the method of Barrs and Weatherley [38]. LWC was calculated as a percentage using the formula:

$$\:LWC\:\left(\%\right)\:=\:\left[\right(FW\:-\:DW)/\:(TW\:-\:DW\left)\right]\:\times\:\:100$$

where FW represents fresh weight, DW represents dry weight (obtained after drying samples at 70 °C for 72 h), and TW represents turgid weight (obtained after saturating samples in distilled water at 4 °C for 24 h).

The total chlorophyll (TCh) concentration was quantified using a SPAD-502Plus meter (Konica Minolta, Japan). The SPAD readings were transformed into real chlorophyll content utilizing the calibration equation established by Uddling et al. [39] as follows: TCh (mg/g FW) = 0.0996 × SPAD value + 0.152.

The photosynthetic rate (PhR) and transpiration rate (TR) were assessed utilizing a LI-6400XT Portable Photosynthesis System (LI-COR Inc., USA), in accordance with the methodologies outlined by von Caemmerer and Farquhar [40]. Measurements were performed on the ear leaf from 09:00 to 11:00 h under regulated circumstances. The system was sustained at a photosynthetic photon flux density of 1500 µmol m⁻² s⁻¹, a leaf chamber temperature of 25 ± 1 °C, a CO₂ concentration of 400 ± 5 µmol mol⁻¹, and a relative humidity of 60 ± 5%.

#### Biochemical analysis (Oxidative stress Markers)

The concentration of hydrogen peroxide (H₂O₂) was assessed using the methodology established by Velikova et al. [41]. Fresh leaf tissue (0.5 g) was homogenized in 5 mL of 0.1% (w/v) trichloroacetic acid and subsequently centrifuged at 12,000 g for 15 min. A 0.5 mL portion of the supernatant was combined with 0.5 mL of 10 mM potassium phosphate buffer at pH 7.0 and 1 mL of 1 M KI. Absorbance was quantified spectrophotometrically at 390 nm, and hydrogen peroxide concentration was determined utilizing a standard curve.

The concentration of superoxide anion (O₂⁻) was evaluated utilizing the nitroblue tetrazolium (NBT) reduction technique as outlined by Doke [42]. Leaf discs (1 cm²) were immersed in 10 mM potassium phosphate buffer (pH 7.8) with 0.05% NBT and incubated for 1 h under illuminated conditions at 25 °C. The samples were subsequently heated to 85 °C in ethanol for 15 min, and the absorbance was recorded at 580 nm.

The malondialdehyde (MDA) concentration was assessed using the thiobarbituric acid reaction technique outlined by Heath and Packer [43]. Fresh tissue samples (0.5 g) were homogenized in 5 mL of 0.1% trichloroacetic acid and subsequently centrifuged at 10,000 g for 5 min. One milliliter of the supernatant was combined with 4 mL of 20% trichloroacetic acid containing 0.5% thiobarbituric acid. The amalgamation was subjected to heating at 95 °C for 30 min, followed by rapid cooling in an ice bath. Following centrifugation at 10,000 g for 10 min, the absorbance of the supernatant was assessed at 532 nm and 600 nm. The MDA concentration was determined utilizing an extinction value of 155 mM⁻¹cm⁻¹.

#### Yield components

Yield components were evaluated at harvest (115 DAS). The number of cobs per square meter (NCM) was assessed by counting the cobs in a 1 m² sample area. The number of grains per line of cob (NGL) was determined by averaging counts from ten randomly selected cobs. The hundred-grain weight (HGW) was calculated by averaging four distinct samples. Grain yield (GY) was derived from the harvest of central rows, whereas biological yield (BY) was assessed as the total above-ground dry biomass.

### Statistical analysis

The data were analyzed using analysis of variance (ANOVA) in a split-split-plot design arranged in a randomized complete block design (RCBD) with three replications. Irrigation methods were assigned to the main plots, mulching treatments to the sub-plots, and foliar applications to the sub-sub-plots. Statistical analyses and visualizations were conducted in RStudio (Version 2023.12.1) with R version 4.3.2 (R Core Team, 2023). The experimental design analysis was performed using the “agricolae” package (version 1.3.7), including ANOVA and multiple comparisons of means via Tukey’s HSD test, with significance set at *p* ≤ 0.05.

Relationships among the measured traits and the main sources of variation in the dataset were investigated through principal component analysis (PCA) using the “factoextra” and “FactoMineR” packages. A clustering heatmap was generated using the “pheatmap” package (version 1.0.12) to visualize treatment performance across different traits and illustrate the relationships between traits and treatments. Structural equation modeling (SEM) was carried out using the “lavaan” package (version 0.6.17) to explore hypothesized causal relationships between irrigation methods, mulching, foliar applications, and their direct effects on growth, yield, and physiological parameters. Data manipulation was performed using the “tidyverse” suite (version 2.0.0), while visualizations were generated using “ggplot2” (version 3.4.4) for basic plots, “corrplot” for correlation analysis, and “fmsb” for radar plot construction.

## Results

### ANOVA showing effects of irrigation, mulching, and foliar spray on maize growth, yield, and physiological traits

The analysis of variance revealed significant effects of irrigation systems, mulching, and foliar spray treatments, as well as their interactions on maize growth parameters, yield components, and physiological traits. In the first season (Table 2), the main effects of irrigation systems significantly (*p* ≤ 0.001) influenced growth parameters, including plant height (PH), net assimilation rate (NAR), crop growth rate (CGR), and leaf area index (LAI). Similarly, mulching and foliar spray treatments showed significant effects on these growth parameters. Regarding yield components, significant responses were observed for number of cobs per m² (NCM), number of grains per line of cob (NGL), 100-grain weight (HGW), grain yield (GY), and biological yield (BY) under all three main factors. The mulching treatment exhibited the strongest effect on NGL and BY, while irrigation systems markedly influenced GY.

Physiological traits including leaf water content (LWC), total chlorophyll (TCh), photosynthetic rate (PhR), transpiration rate (TR), in addition to stress indicators parameters (hydrogen peroxide (H_2_O_2_), superoxide anion (O2˙‾), and malondialdehyde activity (MDA)) were significantly affected by all treatments. Notable effects were observed in LWC and MDA under both irrigation and spray treatments. The interaction effects between these factors were also significant. The irrigation × mulching interaction significantly affected most parameters, particularly yield components and physiological traits. The irrigation × spray interaction showed significant effects on growth parameters and yield components. The three-way interaction (irrigation × mulching × spray) was significant for most measured parameters.

The second season (Table 3) showed similar trends to the first season in terms of the significant effects of irrigation systems, mulching, and foliar spray treatments, as well as their interactions on maize growth parameters, yield components and physiological traits. In growth parameters, while the irrigation systems maintained their significant influence (*p* ≤ 0.001) on PH, NAR, and CGR, the CGR showed a more pronounced response to treatments compared to the first season. For yield components, the patterns remained consistent with the first season, but the mulching treatment demonstrated a stronger effect on HGW and GY in the second season, while irrigation systems showed an enhanced influence on BY. Regarding physiological traits, while the overall significant effects persisted, LWC and PhR exhibited more prominent responses to irrigation and spray treatments compared to the first season. The interaction effects maintained their significance, with irrigation × mulching interaction showing particularly strong effects on PhR and MDA in the second season. These results pinpoint the findings from the first season while highlighting some seasonal variations in the magnitude of responses to the treatments.

**Table 2:** Analysis of variance (ANOVA) for growth, yield, and physiological parameters of maize under irrigation, mulching, and foliar spray treatments in the first season of study

**Source**	**Df**	**PH**	**NAR**	**CGR**	**NCM**	**NGL**	**HGW**	**GY**	**BY**
Block	2	0.0006	1.08	0.89	0.03	0.17ns	3.7	0.01	2.65
IR	2	0.86***	164.26***	1795.27***	1.36***	85.74***	21.53**	11.61***	188.15***
Error (a)	4	0.0002	0.07	0.34	0.02	0.3	0.4	0.002	0.17
Mulch	1	0.68***	69.52***	438.80***	2.02***	1046.32***	444.33***	9.85***	947.02***
IR x Mulch	2	0.005**	1.52**	64.46***	0.001ns	4.88**	39.44***	0.53***	9.77*
Error (b)	6	0.0002	0.07	0.51	0.02	0.28	0.51	0.01	1.15
Spray	2	0.63***	161.02***	4315.11***	3.07***	320.24***	30.20***	20.90***	959.85***
IR x Spray	4	0.009***	4.46***	210.49***	0.11***	2.15**	60.13***	0.32***	17.85***
Mulch x Spray	2	0.002*	1.94***	2.02*	0.06*	18.91***	63.27***	0.04**	9.34**
IR x Mulch x Spray	4	0.01***	9.80***	3.06**	0.04ns	5.26**	42.49***	0.08***	5.45**
Error (c)	24	0.0004	0.1	0.48	0.02	0.44	0.87	0.01	1.02
**Source**	**Df**	**LAI**	**LWC**	**TCh**	**PhR**	**TR**	**H**_**2**_ **O**_**2**_	**O2˙‾**	**MDA**
Block	2	0.0006	1.28	0.004	1.57	0.0003	0.002	0.0001	0.16
IR	2	1.05***	721.01***	0.48***	264.35***	2.26***	88.33***	0.25***	812.91***
Error (a)	4	0.0002	0.24	0.005	1.6	0.0003	0.002	0	0.02
Mulch	1	0.50***	282.91***	0.42***	53.72**	0.11***	8.22***	0.17***	141.91***
IR x Mulch	2	0.01***	19.75***	0.12***	2.42ns	0.05***	0.58***	0.14***	0.87**
Error (b)	6	0.0006	1.24	0.003	1.99	0.0002	0.003	0	0.07
Spray	2	3.26***	1039.42***	3.32***	94.44***	1.14***	21.55***	0.21***	264.39***
IR x Spray	4	0.05***	23.62***	0.04***	6.03ns	0.44***	0.24***	0.15***	10.57***
Mulch x Spray	2	0.03***	29.38***	0.07***	3.28ns	0.008***	0.52***	0.14***	9.19***
IR x Mulch x Spray	4	0.003***	7.67***	0.005***	5.14ns	0.006***	0.10***	0.14***	1.91***
Error (c)	24	0.0005	1.01	0.004	2.21	0.0006	0.001	0	0.05

PH: Plant height, NAR: Net assimilation rate, CGR: Crop growth rate, NCM: Number of cobs per m², NGL: Number of grains per line of cob, HGW: 100-grain weight, GY: Grain yield, BY: Biological yield, LAI: Leaf area index, LWC: Leaf water content, TCh: Total chlorophyll, PhR: Photosynthetic rate, TR: Transpiration rate, H_2_O_2_: Hydrogen peroxide, O2˙‾: Superoxide anion, MDA: Malondialdehyde activity. *, **, *** = Significant at *p* ≤ 0.05, *p* ≤ 0.01, and *p* ≤ 0.001, respectively. ns = non-significant

**Table 3:** Analysis of variance (ANOVA) for growth, yield, and physiological parameters of maize under irrigation, mulching, and foliar spray treatments in the second season of study

**Source**	**Df**	**PH**	**NAR**	**CGR**	**NCM**	**NGL**	**HGW**	**GY**	**BY**
Block	2	0.0004	0.0002ns	0.0125	0.0015	0.3215	3.7006	0.0131	0.4338
IR	2	0.35***	137.98***	4767.07***	0.79***	147.80***	21.53**	7.90***	1070.20***
Error (a)	4	0.0002	0.002	0.1	0.0008	0.08	0.4	0.01	0.59
Mulch	1	0.87***	21.43***	42.01***	4.67***	181.10***	444.33***	45.34***	290.79***
IR x Mulch	2	0.006*	0.002ns	0.13*	0.44***	9.31***	39.44***	41.30***	3.40***
Error (b)	6	0.0005	0.002	0.02	0.001	0.05	0.51	0.03	0.05
Spray	2	1.59***	336.78***	3852.76***	9.96***	279.20***	30.2***	25.65***	2226.98***
IR x Spray	4	0.005***	0.54***	213.44***	0.09***	3.89***	60.13***	0.49***	45.71***
Mulch x Spray	2	0.034***	0.01***	0.90***	0.04***	1.55***	63.27***	2.08***	13.22
IR x Mulch x Spray	4	0.002**	0.002ns	0.49**	0.01***	2.48***	42.49***	0.76***	8.12***
Error (c)	24	0.0003	0.001	0.08	0.002	0.08	0.87	0.03	0.25
**Source**	**Df**	**LAI**	**LWC**	**TCh**	**PhR**	**TR**	**H**_**2**_ **O**_**2**_	**O2˙‾**	**MDA**
Block	2	0.001	0.8734	0.0001	0.0211	0.0012	0.04	0	0.02
IR	2	1.15***	1550.69***	0.018**	4.23***	0.02***	0.53*	0.15***	8.58***
Error (a)	4	0.001	0.45	0.0004	0.05	0.0004	0.03	0	0.14
Mulch	1	0.67***	910.37***	1.86***	5.10***	0.002*	0.17*	0.0005**	2.67**
IR x Mulch	2	0.08***	32.61**	1.15***	83.25***	0.46***	27.05***	0.64***	225.24***
Error (b)	6	0.001	1.76	0.0002	0.03	0.0002	0.02	0	0.12
Spray	2	5.33***	776.90***	1.46***	147.93***	0.90***	76.85***	1.009***	348.70***
IR x Spray	4	0.04***	2.48**	0.008***	1.49***	0.09***	0.31***	0.14***	2.94***
Mulch x Spray	2	0.001**	8.34***	0.045***	0.05*	0.21***	0.31**	0.001***	0.89**
IR x Mulch x Spray	4	0.04***	5.93***	0.03***	2.46***	0.14***	0.24***	0.36***	7.81***
Error (c)	24	0.0004	0.56	0.0003	0.02	0.0003	0.04	0	0.12

PH: Plant height, NAR: Net assimilation rate, CGR: Crop growth rate, NCM: Number of cobs per m², NGL: Number of grains per line of cob, HGW: 100-grain weight, GY: Grain yield, BY: Biological yield, LAI: Leaf area index, LWC: Leaf water content, TCh: Total chlorophyll, PhR: Photosynthetic rate, TR: Transpiration rate, H_2_O_2_: Hydrogen peroxide, O2˙‾: Superoxide anion, MDA: Malondialdehyde activity. *, **, *** = Significant at *p* ≤ 0.05, *p* ≤ 0.01, and *p* ≤ 0.001, respectively. ns = non-significant

### Growth, yield components, physiological response, and stress indicators parameters of maize under different combined irrigation systems, foliar applications, and mulching treatments

**Fig. 2:**
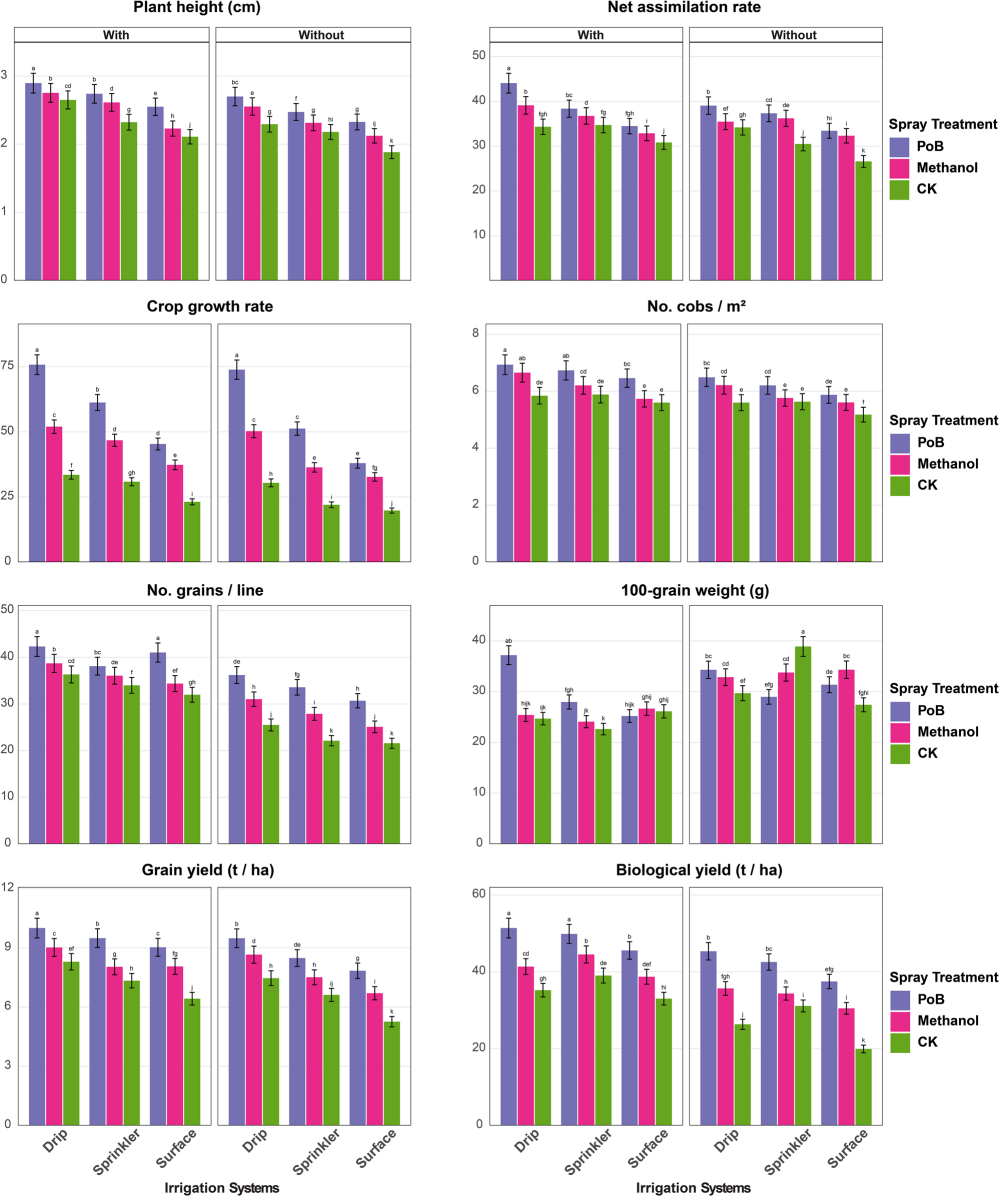

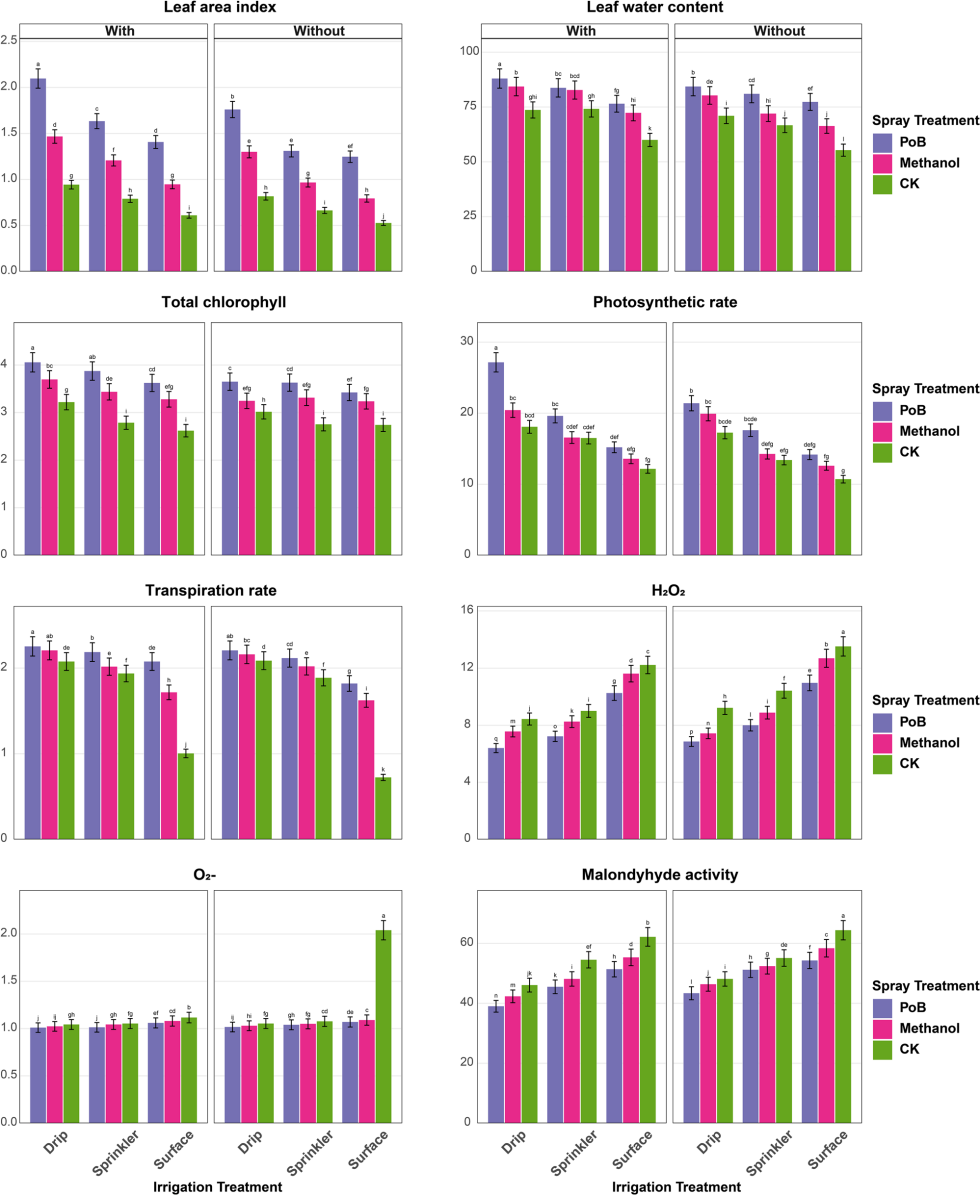
**a**. Growth parameters, yield components, and physiological traits of maize under different irrigation systems (surface, sprinkler, drip) and foliar spray treatments (PoB: potassium bicarbonate, Methanol, CK: control), with and without mulching in the first season. Error bars represent standard error. Different letters above bars indicate significant differences between treatments at *p* ≤ 0.05 According to the Tukey–Kramer Honest Significant Difference (HSD) test. **b** Growth parameters, yield components, and physiological traits of maize under different irrigation systems (surface, sprinkler, drip) and foliar spray treatments (PoB: potassium bicarbonate, Methanol, CK: control), with and without mulching in the first season. Error bars represent standard error. Different letters above bars indicate significant differences between treatments at *p* ≤ 0.05 According to the Tukey–Kramer Honest Significant Difference (HSD) test

**Fig. 3:**
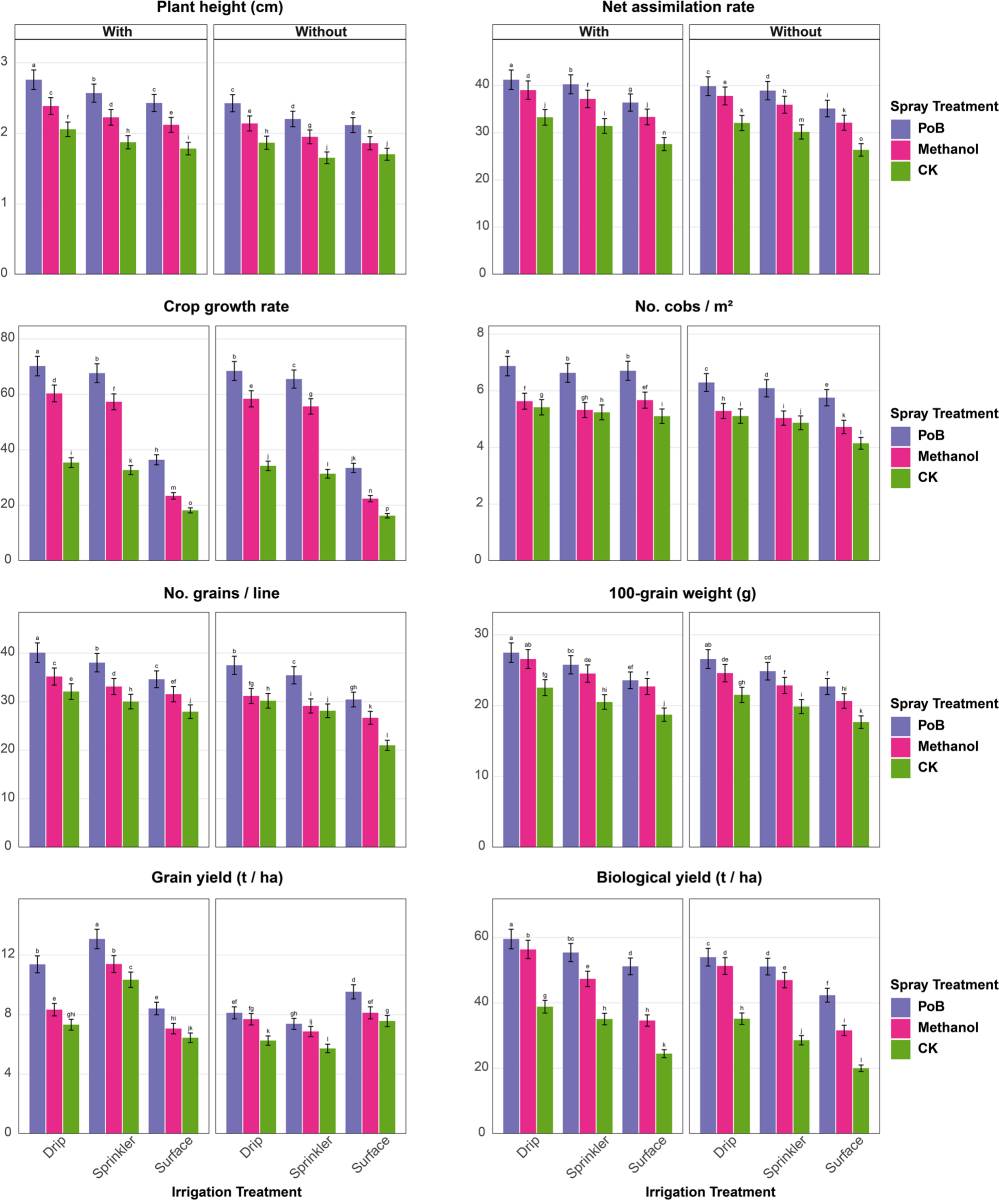

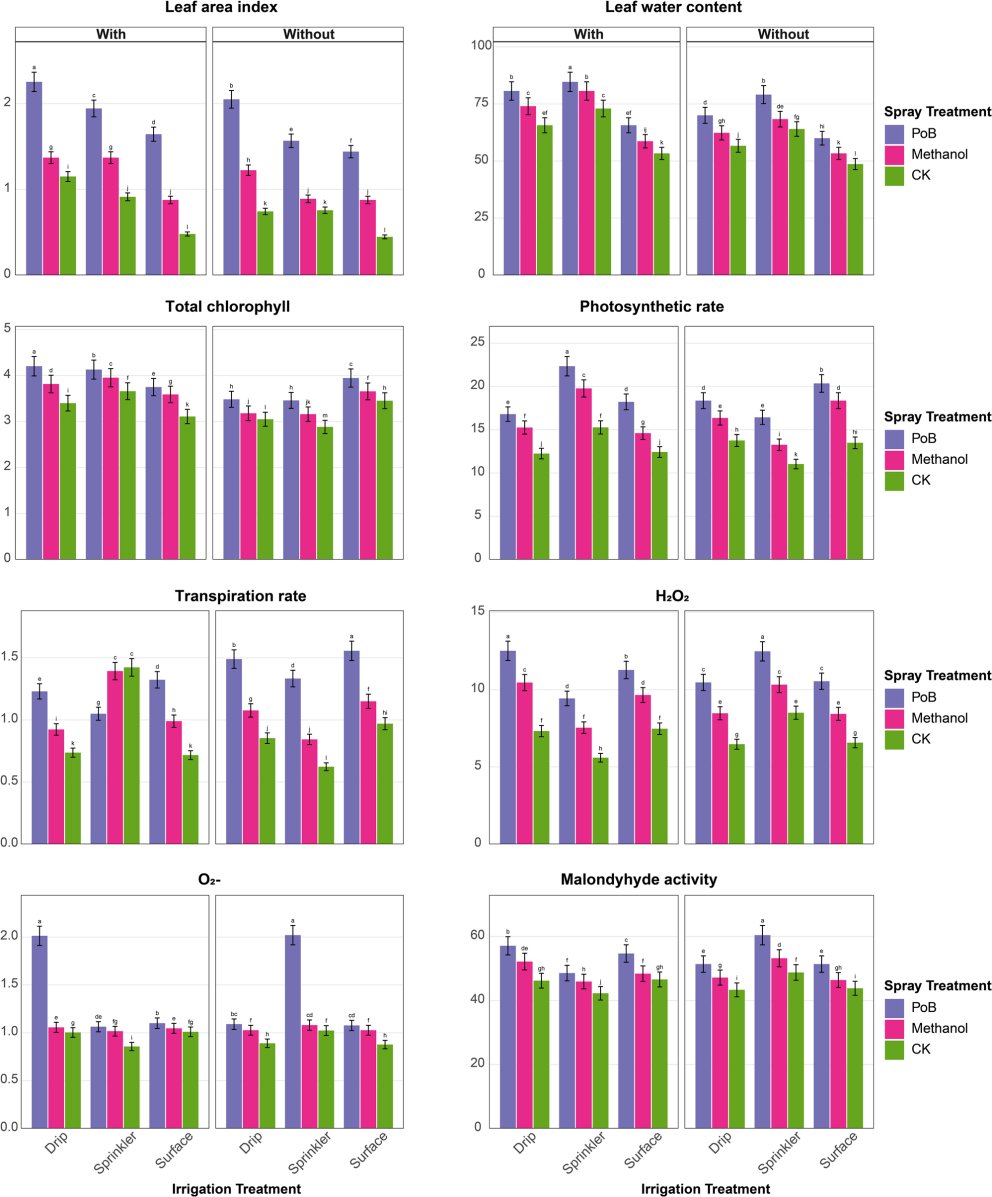
**a**. Growth parameters, yield components, and physiological traits of maize under different irrigation systems (surface, sprinkler, drip) and foliar spray treatments (PoB: potassium bicarbonate, Methanol, CK: control), with and without mulching in the second season. Error bars represent standard error. Different letters above bars indicate significant differences between treatments at *p* ≤ 0.05 According to the Tukey–Kramer Honest Significant Difference (HSD) test. **b**. Growth parameters, yield components, and physiological traits of maize under different irrigation systems (surface, sprinkler, drip) and foliar spray treatments (PoB: potassium bicarbonate, Methanol, CK: control), with and without mulching in the second season. Error bars represent standard error. Different letters above bars indicate significant differences between treatments at *p* ≤ 0.05 According to the Tukey–Kramer Honest Significant Difference (HSD) test

The findings indicated significant influences of irrigation systems, foliar applications, and mulching on the growth and physiological performance of maize across both the first season (Fig. 2) and the second season (Fig. 3).

In the first season (Fig. 2a-b), the results showed that PH had the highest values under drip irrigation with potassium bicarbonate (PoB) and mulching (2.89 m), while the lowest value was recorded under surface irrigation without control treatment (CK) and mulching (1.88 m). Also, NAR followed similarly, with Drip_PoB_mulching yielding the highest NAR (44.08 g/cm²/day), versus surface-CK-mulching (26.59 g/cm²/day). The CGR peaked under Drip_PoB_mulching (75.76 g/cm²/day), significantly exceeding surface-CK-mulching (19.79 g/cm²/day, Fig. 2a). The NCM was the highest with treatments combination of Drip_PoB_mulching (6.93), whereas surface-CK-mulching produced the lowest (5.18). The NGL showed superior results under Drip_PoB_mulching (42.33), contrasting with surface-CK-mulching (21.58). For GY, the treatment combination of Drip_PoB_mulching had the most enhanced level of GY (10 t/ha), while surface-CK-mulching yielded the lowest level (5.26 t/ha). Similarly, BY had its maximal levels (51.37 t/ha) with Drip_PoB_mulching, however, the surface-CK-mulching yielded the lowest (19.91 t/ha, Fig. 2a).

Physiological parameters showed significant responses (Fig. 2b), as LWC was highest under Drip_PoB_mulching (88%), versus surface-CK-mulching (55.33%). LAI had the highest level under the combined treatment of Drip_PoB_mulching (2.10%), while surface-CK-mulching showed the lowest (0.53%). The levels of TCh peaked under Drip_PoB_mulching (4.06 mg g⁻¹ FW), while surface_CK_mulching showed the lowest (2.62 mg g⁻¹ FW). Also, PhR maximized under Drip_PoB_mulching (27.16 µmol CO₂ m⁻²s⁻¹), significantly exceeding surface-CK-mulching (10.72 µmol CO₂ m⁻²s⁻¹). Notably, the stress indicators showed inverse patterns. H₂O₂ content was lowest under Drip_PoB_mulching (6.40 µmol g⁻¹ FW), while surface-CK-mulching showed the highest value of 13.53 µmol g⁻¹ FW. Similarly, O₂⁻ levels were the lowest under Drip_PoB_mulching (1.01 µmol g⁻¹ FW), contrasting with surface-CK-mulching (2.04 µmol g⁻¹ FW). Furthermore, the activity of MDA activity showed the same trend, lowest under Drip_PoB_mulching (39.00 nmol g⁻¹ FW), and highest under surface-CK-mulching (64.38 nmol g⁻¹ FW, Fig. 2b).

In the second season (Fig. 3a-b), PH peaked under Drip_PoB_mulching (2.76 m), whereas it was the lowest under the treatment of sprinkler-CK-mulching (1.65 m). NAR was highest with Drip_PoB_mulching (41.21 g/cm²/day), minimum under surface-CK-mulching (26.35 g/cm²/day). CGR maximized under Drip_PoB_mulching (70.20 g/cm²/day), while surface-CK-mulching showed lowest (16.24 g/cm²/day). NCM was highest with Drip_PoB_mulching (6.87), and lowest under surface-CK-mulching (4.15). NGL showed the best results under Drip_PoB_mulching (40.08), contrasting with surface-CK-mulching (21.00). HGW was highest under Drip_PoB_mulching (27.48 g), and lowest with surface-CK-mulching (17.68 g). GY peaked under sprinkler_PoB_mulching (13.08 t/ha), the minimum under sprinkler-CK-mulching (5.73 t/ha). BY was maximum with Drip_PoB_mulching (59.53 t/ha), and minimum under surface-CK-mulching (20.00 t/ha, (Fig. 3a).

LAI showed highest values under Drip_PoB_mulching (2.25), lowest under surface-CK-mulching (0.45). LWC maximized under sprinkler_PoB_mulching (84.67%), minimum with surface-CK-mulching (48.67%). TCh peaked under Drip_PoB_mulching (4.20 mg g⁻¹ FW), lowest under sprinkler-CK-mulching (2.88 mg g⁻¹ FW). PhR was highest with sprinkler_PoB_mulching (22.35 µmol CO₂ m⁻²s⁻¹), minimum under sprinkler-CK-mulching (11.04 µmol CO₂ m⁻²s⁻¹). H₂O₂ and O₂⁻ were highest under Drip_PoB_mulching (12.51 and 2.01 µmol g⁻¹ FW) and lowest under sprinkler_CK_mulching (5.59 and 0.86 µmol g⁻¹ FW). MDA was highest under sprinkler-PoB-mulching (60.43 nmol g⁻¹ FW), and lowest with sprinkler_CK_mulching (42.27 nmol g⁻¹ FW, (Fig. 3b)

Interestingly, comparative analysis between the two seasons revealed distinct patterns in maize response to treatments. Growth parameters showed superior performance in the first season, with PH, NAR, and CGR showing increases of 4.7%, 7.0%, and 7.9% respectively compared to the second season under optimal conditions (Drip_PoB_mulching). Yield components demonstrated contrasting trends, with the second season showing improvements. GY increased by 30.8% (from 10.0 to 13.08 t/ha) and BY improved by 15.9% (from 51.37 to 59.53 t/ha) under optimal treatments. However, NCM and NGL maintained slightly higher values in the first season, exceeding the second season by 0.9% and 5.6% respectively. Physiological parameters showed mixed responses between seasons. While LWC and PhR were higher in the first season by 3.9% and 21.5% respectively, TCh showed a modest increase of 3.4% in the second season. LAI demonstrated comparable values across both seasons under optimal conditions. Stress indicators exhibited notable seasonal variations. The second season showed higher oxidative stress markers, with H₂O₂ and O₂⁻ levels increasing by 95.5% and 99% respectively compared to the first season. However, MDA decreased by 34.3% in the second season, suggesting potential stress adaptation mechanisms.

### Hierarchical analysis of treatment combinations and their effects on maize physiological and growth attributes

**Fig. 4:**
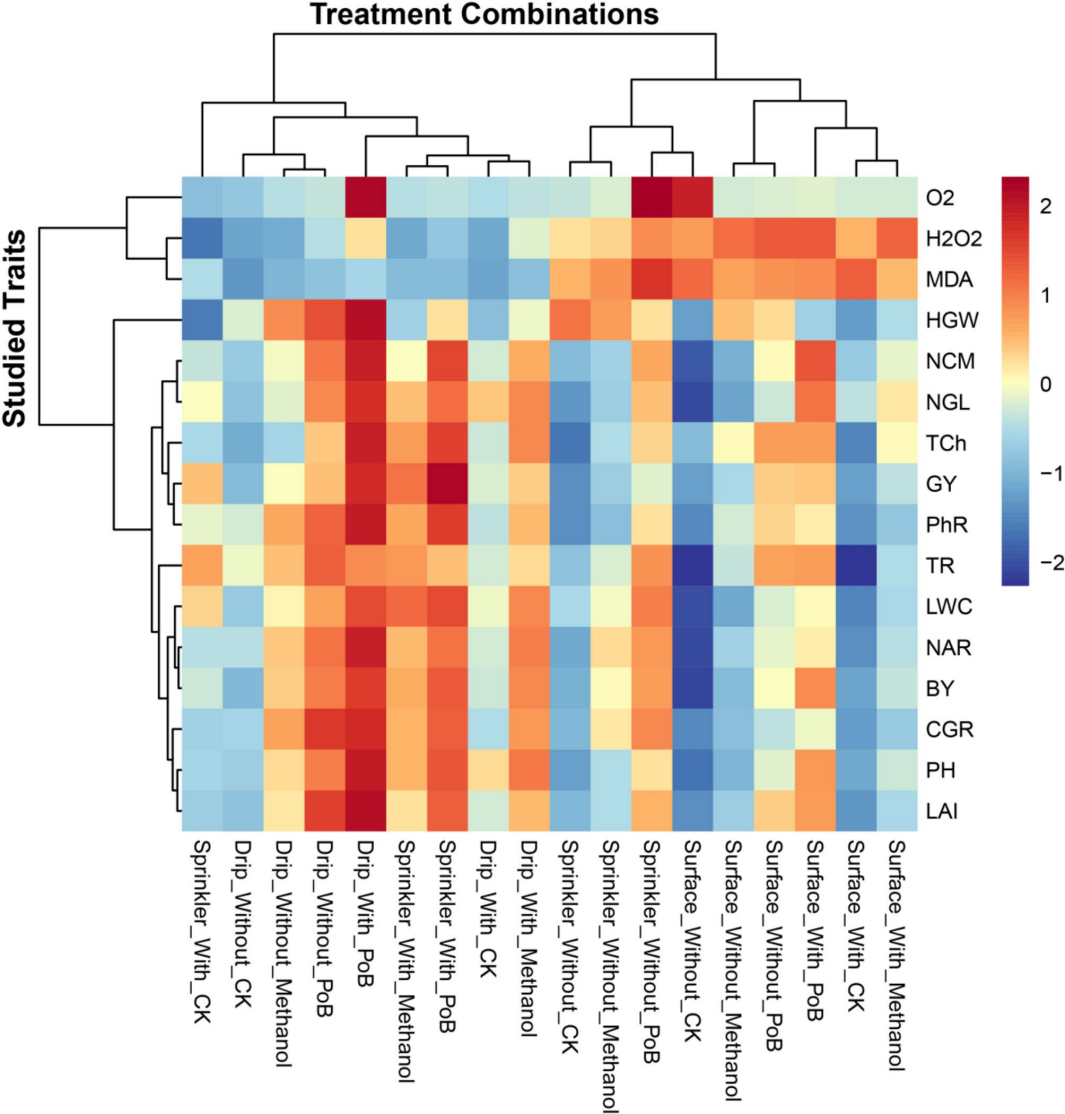
Hierarchical clustering heatmap showing interactions between irrigation systems (drip, sprinkler, and surface), mulching treatments (with and without), and foliar applications (CK, methanol, and PoB) on 16 growth, yield, physiological traits of maize. The color scale represents standardized values ranging from − 2 (dark blue) to 2 (dark red). Dendrograms on both axes indicate clustering patterns among treatments and traits. PH: Plant height (cm), NAR: Net assimilation rate, CGR: Crop growth rate, NCM: No. cobs / m², NGL: No. grains/line of cob, HGW: 100-grain weight (g), GY: Grain yield (t/ha), BY: Biological yield (t/ha), LAI: Leaf area index, LWC: Leaf water content, TCh: Total chlorophyll, PhR: Photosynthetic rate, TR: Transpiration rate, H_2_O_2_: Hydrogen peroxide, O2˙‾: Superoxide anion, MDA: Malondyhyde activity

The clustering heatmap analysis (Fig. 4) revealed distinct patterns in treatment-trait relationships for maize growth, physiology, and productivity. Traits, including PH, LAI, and CGR, demonstrated peak performance under drip irrigation with mulching and PoB treatments. LAI measurements were notably enhanced when drip irrigation was combined with mulching and PoB applications, showing marked improvements compared to surface irrigation methods. PH and CGR exhibited similar response patterns under these optimal irrigation conditions. Also, HGW, NCM, and NGL parameters clustered together, exhibiting maximum responses under drip irrigation with mulching and PoB combinations. NCM and NGL reached their highest values under these conditions, while HGW showed similar positive responses.

Traits, including TCh, GY, and PhR formed a distinct cluster and showed the strongest positive responses to drip and sprinkler treatments combined with mulching and PoB. TR decreased under mulching treatments, particularly with drip irrigation systems. LWC and NAR demonstrated similar response patterns under drip irrigation with mulching and PoB treatments. BY showed a strong positive correlation with these parameters under the same treatment combinations. The stress indicator parameters of O2˙‾, H_2_O_2_, and MDA noticeably clustered together, showing the highest intensities under treatments without mulching, particularly in surface irrigation combinations. Surface irrigation without mulching consistently produced the lowest responses across all measured parameters, particularly when combined with control treatments. Adding mulching improved performance across all irrigation systems, but the improvement was most pronounced in drip irrigation.

Overall, the dendrograms revealed distinct treatment clusters: drip with mulching_PoB showing superior results, followed by sprinkler with mulching_PoB and drip with mulching_methanol, then surface with mulching_PoB and sprinkler with mulching_methanol, and finally surface without mulching_CK showing the weakest performance. These clusters demonstrated clear separation in this hierarchical analysis, with mulching consistently improving performance across all irrigation methods and foliar applications.

### Radar plot analysis of individual effects of treatments on maize traits

**Fig. 5:**
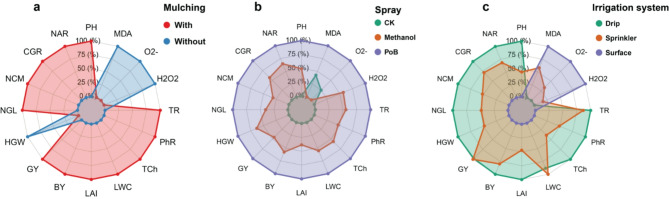
Radar plot represents the effects of different treatments on maize traits. (**a**) Comparison of mulching treatments with (red line) and without (blue line) mulching, highlighting relative contributions to various growth, yield, and stress parameters. (**b**) Effects of foliar spray applications, including potassium bicarbonate (PoB, purple line), methanol (orange line), and the control (CK, green line), with varying contributions across parameters. (**c**) Analysis of irrigation systems: drip irrigation (green line), sprinkler irrigation (orange line), and surface irrigation (purple line), illustrating their contributions to growth, yield, and stress-related traits. Values range from 0% at the center to 100% at the outer edge, with color-coded lines representing the contributions of each treatment

The radar plot analysis revealed distinct patterns in the relative contributions of the treatments studied to maize traits (Fig. 5a-c). The mulching effect (Fig. 5a) demonstrated that treatments with mulching had notably higher contributions to most growth and physiological parameters, particularly for most yield and physiological parameters such as GY, BY, LWC, LAI, LWC, TR, TCh, and PhR, where the contribution reached approximately 75–80% compared to without mulching treatments. Interestingly, for oxidative stress indicators (MDA, O2˙‾, and H_2_O_2_), the pattern was reversed, with treatments without mulching showing higher contributions, reaching approximately 70–75%.

The foliar spray applications comparison (Fig. 5b) illustrated that potassium bicarbonate (PoB) spray (purple line) consistently demonstrated the highest relative contribution across almost all parameters, with values reaching 80–90% for most traits. Methanol spray (orange line) showed intermediate contributions, typically ranging from 40 to 60% across various traits, while the control treatment (CK, green line) exhibited the lowest relative contribution, generally remaining below 30% for most parameters, except for some stress-related traits where it showed slightly higher contributions.

Regarding irrigation systems (Fig. 5c), drip irrigation (green line) dominated in its contribution to growth and yield-related traits, particularly for NAR, CGR, NCM, and NGL, where its contribution reached 75–85%. Sprinkler irrigation (orange line) showed moderate contributions, generally ranging from 40 to 60%, with notably higher contributions to certain traits like PhR and TR. Surface irrigation (purple line) generally showed the lowest contributions to growth and yield parameters, though it exhibited higher contributions to stress-related parameters (MDA, O2˙‾, H_2_O_2_), reaching 60–70% for these traits.

### Principal component analysis of maize growth, yield, physiological and oxidative stress parameters under combined irrigation systems, sprays and mulching treatments

**Fig. 6:**
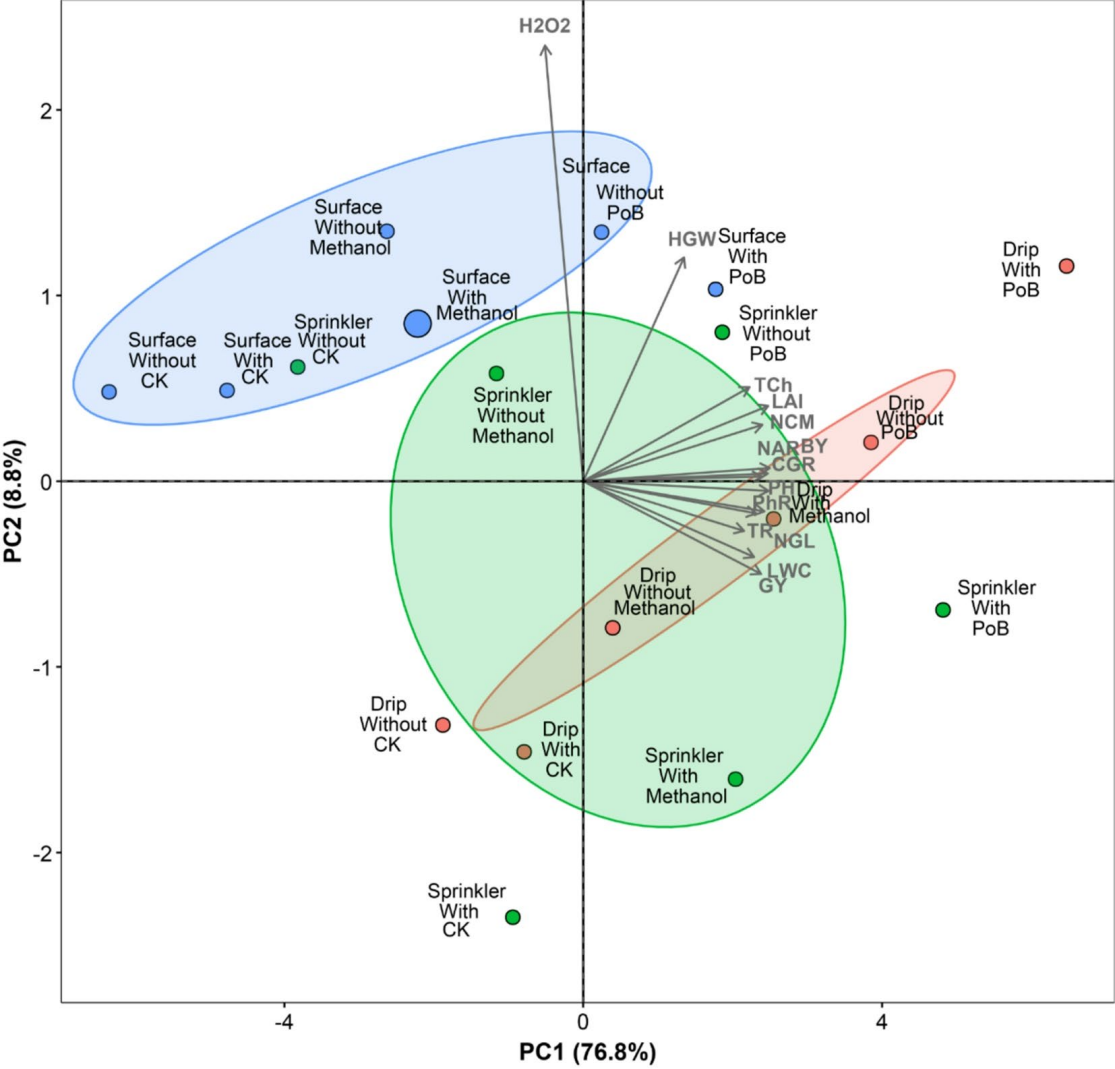
Principal Component Analysis (PCA) biplot showing the relationship between maize traits (growth, yield, physiological and oxidative stress parameters) and treatment combinations of irrigation systems (surface, sprinkler, drip), sprays (ck, methanol, PoB), and mulching (with and without). The biplot explains 85.6% of the total variation (PC1: 76.8%, PC2: 8.8%). Vectors represent physiological traits, while points represent treatment combinations. Colored ellipses indicate the clustering of treatments by irrigation system (blue: surface, green: sprinkler, red: drip). Treatment labels indicate combinations of irrigation method, spray treatment, and mulching status. Vector length and direction indicate the strength and relationship of traits with treatment combinations

The principal component analysis (PCA) revealed that the first two principal components explained a substantial portion of the total variance in the dataset, with PC1 accounting for 76.8% and PC2 explaining 8.8%, collectively capturing 85.6% of the total variation in the measured maize parameters (Fig. 6). The results demonstrated that most growth parameters (such as PH), yield components (such as BY), and physiological traits (such as LAI, NAR, CGR) exhibited strong positive correlations with PC1. Specifically, BY, PH, LAI, and NAR showed the highest loadings on PC1. Interestingly, the oxidative stress indicator H_2_O_2_ displayed a contrasting pattern with a weak negative loading on PC1 (-0.201) but a strong positive loading on PC2 (0.92), indicating its unique response to the treatments.

Notably, the biplot visualization clearly depicted three distinct clusters based on irrigation systems, while within each cluster, the spray treatments and mulching options created sub-patterns. This three-way interaction suggests that the effectiveness of sprays and mulching is dependent on the irrigation system used, with some combinations performing notably better than others in terms of their effects on measured parameters. This comprehensive analysis indicates that while irrigation systems create the primary separation in treatment effects, the interaction with sprays and mulching status creates complex response patterns in maize parameters. The most favorable responses for growth, yield, and physiological parameters generally appeared in treatments combining PoB sprays with mulching, though the magnitude of this effect varied across irrigation systems.

For the treatment effects and pattern distribution, the treatment combinations revealed distinctive patterns across all three combined factors. Regarding irrigation systems, surface irrigation treatments generally clustered in the upper quadrants of the biplot, while sprinkler and drip systems showed distinct groupings in other regions. The foliar spray treatments showed clear differentiation, with PoB treatments (regardless of irrigation system or mulching status) consistently showing positive values on PC1, indicating their positive association with growth and yield parameters. The CK treatments typically displayed negative values on both PC1 and PC2, particularly evident in Surface_Without_CK (-6.346 on PC1). Also, the mulching factor’s effect was evident in the separation of treatments within each irrigation-spray combination. Treatments “with” mulching generally showed better association with growth, yield, and physiological parameters compared to their “without” counterparts, though this effect varied depending on the irrigation system and spray type used.

### Causal relationships between growth, physiology, yield-related traits, oxidative indicator parameters on grain yield of maize under different management practices

**Fig. 7:**
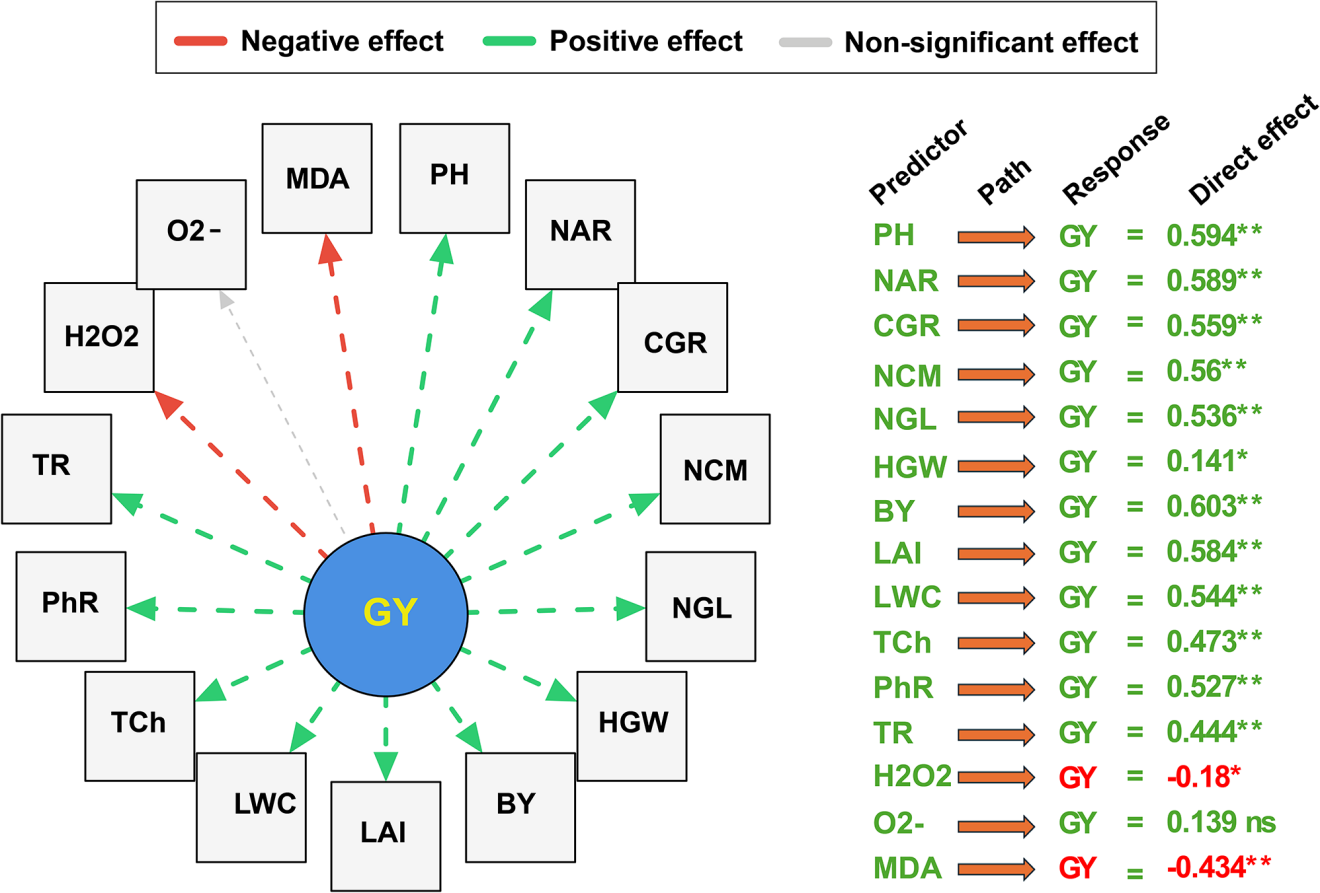
Structural equation model (SEM) showing direct effects of growth, yield-components, physiological, Oxidative indicator traits on maize grain yield (GY). Green and red dashed arrows indicate positive and negative effects, respectively. The Gray dashed arrow represents a non-significant relationship. The numbers in the table represent standardized path coefficients. ** and * indicate significance at *p* ≤ 0.01 and *p* ≤ 0.05, respectively; ns = non-significant

In this study, structural equation modeling (SEM) was employed to quantify the direct effects of the measured traits (as predictors) on GY under different irrigation systems, foliar applications, and mulching treatments (Fig. 7). The results revealed significant causal relationships among the studied variables. Overall, BY showed the strongest positive effect on GY (0.603), followed by PH (0.594) and NAR (0.589). Also, CGR (0.559), NCM (0.56), and NGL (0.536) also demonstrated significant positive effects, highlighting the importance of reproductive efficiency. The HGW showed a smaller but significant positive effect (0.141).

In addition, physiological traits significantly influenced GY, with LAI and LWC showing strong positive effects (0.584 and 0.544, respectively). In addition, TCh (0.473), PhR (0.527), and TR (0.444) also contributed positively, emphasizing the importance of photosynthetic efficiency and water transport in crop productivity. On contrast, oxidative stress indicators negatively impacted GY. For instance, H₂O₂ showed a significant negative effect (-0.18), while MDA had a stronger negative impact (-0.434), whereas O₂⁻ showed a non-significant effect (0.139). These results demonstrate that maize grain yield is primarily influenced by traits enhancing biomass accumulation and photosynthetic efficiency, while oxidative stress markers negatively affect yield.

### Water use efficiency as affected by individual and combined treatments

**Fig. 8:**
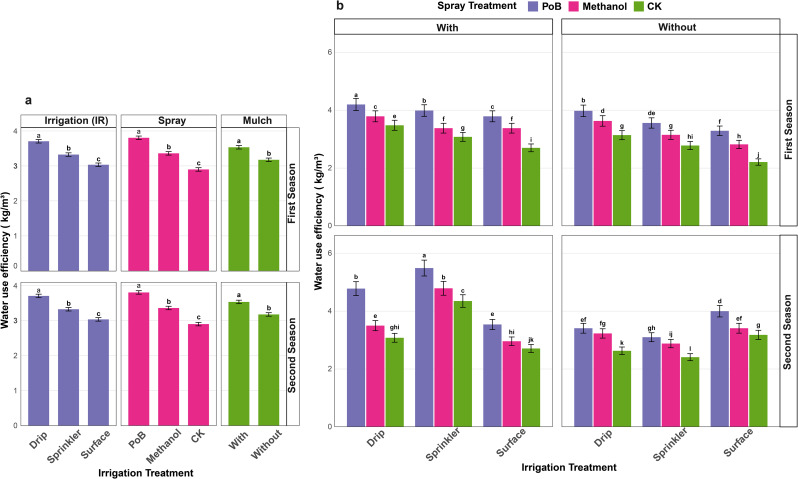
Water use efficiency (WUE) as affected by irrigation methods, mulch application, and spray treatments across two growing seasons. (**a**) Individual effects of irrigation methods (drip, sprinkler, and surface), spray treatments (PoB, Methanol, and CK), and mulch application (with and without) on WUE in first and second seasons. (**b**) Combined effects of irrigation methods under different mulch conditions (with and without) and spray treatments (PoB, Methanol, and CK) on WUE in first and second seasons. Error bars represent standard error of means. Different letters indicate significant differences at *p* ≤ 0.05

Water Use Efficiency (WUE) was substantially affected by irrigation techniques, mulch application, and foliar spray applications during both growth seasons (Fig. 8a). Drip irrigation had the highest water usage efficiency among irrigation systems, with an average value of 3.70 kg/m³, markedly surpassing other techniques. Sprinkler irrigation had moderate performance with an average WUE of 3.32 kg/m³, but surface irrigation shown the least efficiency at 3.03 kg/m³. The comparative efficacy of different irrigation techniques showed persistent statistical significance in both seasons. The utilization of mulch significantly influenced water use efficiency in the experimental plots. Mulched plots had much superior WUE at 3.53 kg/m³, in contrast to non-mulched plots, which recorded 3.17 kg/m³. The beneficial impact of mulching was consistently considerable over both growth seasons. Spray treatments had distinct impacts on water usage efficiency. Foliar application of PoB proved to be the most efficacious, achieving the greatest mean WUE of 3.80 kg/m³. The methanol spray treatment yielded an intermediate WUE of 3.36 kg/m³, whereas the control exhibited the lowest WUE at 2.90 kg/m³.

The interaction effects of irrigation methods, mulch application, and spray treatments demonstrated significant variations in WUE across both growing seasons (Fig. 8b). During the initial season, the integration of drip irrigation with mulch and foliar spray using PoB resulted in the highest WUE at 4.20 kg/m³. This was closely followed by sprinkler irrigation combined with mulch and PoB, which recorded a WUE of 3.99 kg/m³, and drip irrigation without mulch but utilizing PoB, which achieved a WUE of 3.98 kg/m³. The minimum WUE recorded was 2.21 kg/m³ in surface irrigation without mulch and without spray treatment (CK). In the second season, the treatment combinations exhibited varying patterns. Sprinkler irrigation combined with mulch and PoB treatment exhibited the highest WUE at 5.49 kg/m³. In contrast, sprinkler irrigation with mulch and methanol spray, as well as drip irrigation with mulch and PoB, yielded statistically comparable results of 4.79 and 4.78 kg/m³, respectively. The lowest WUE was observed in the sprinkler irrigation treatment without mulch and without spray treatment, measuring 2.41 kg/m³, consistent with findings from the first season.

## Discussion

The findings of this study provide critical insights into the synergistic effects of irrigation systems, mulching, and PoB foliar applications on the growth, yield, and physiological performance of maize in newly reclaimed sandy soils. This research establishes a foundation for improving sustainable agricultural practices in resource-limited environments. The results of the ANOVA demonstrated that irrigation systems, mulching, and PoB foliar spray treatments had substantial impacts on maize growth, yield components, and physiological characteristics. Mulching with rice straw significantly improved yield components, particularly NGL and BY. This is consistent with the findings that mulching improves soil structure, reduces transpiration, and improves soil moisture retention [15, 18]. The synergistic effects of irrigation and mulching are emphasized by their significant interaction, in which mulching enhances the advantages of efficient irrigation by ensuring that the soil conditions are optimal for root development and nutrient availability. Foliar applications with PoB significantly contributed to the improvement of physiological traits including LWC, TCh, and PhR. This aligns with research indicating that PoB mitigates oxidative stress and enhances photosynthetic efficiency through the augmentation of antioxidant enzyme activity [44, 45].

Noteworthy, the results of the current study demonstrated significant seasonal fluctuations, with distinct patterns observed in the relationship between yield components and growth parameters. Although vegetative growth exhibited improved performance in the initial season, yield components exhibited substantial improvement in the subsequent season, despite significantly elevated oxidative stress markers. Environmental factors, including temperature and precipitation patterns, can be responsible for this paradoxical relationship, as they differentially influence reproductive development and vegetative growth [46, 47]. The successful adaptation of the plant to stress conditions is indicated by the diminished MDA levels and the enhanced response of physiological characteristics, including LWC and PhR, in the second season [48, 49]. Under optimal conditions, these adaptive mechanisms resulted in extraordinary yield improvements, including an increase in biomass yield and a 30.8% increase in cereal yield. This suggests that resource use efficiency was significantly improved [50]. These results are consistent with comparable adaptive responses that have been documented in extensive long-term field investigations [51], underscoring the practical implications for maize productivity and sustainable agricultural intensification.

In particular, the integrated management practices applied in this research had a substantial impact on the physiological processes essential for maize growth, development, and yield formation. The combined impacts of drip irrigation, rice straw mulching, and PoB foliar application were clearly observed in increased photosynthetic efficiency, better water relations, and optimized assimilate distribution, all of which together led to enhanced yield characteristics [52]. In this regard, photosynthetic parameters (TCh and PhR) under the optimal treatment combination (drip irrigation_mulching_PoB) can be ascribed to various interrelated physiological mechanisms. Drip irrigation provided a reliable source of soil moisture, thereby sustaining optimal leaf water status and inhibiting stomatal closure during periods of water deficit. This is essential for facilitating CO₂ diffusion into leaves and ensuring elevated photosynthetic rates [53]. The increased leaf water content noted in our study corroborates this interpretation, as sufficient hydration is critical for optimal chloroplast function and enzyme activity within the Calvin cycle [54]. The foliar application of PoB improved photosynthetic efficiency through various pathways. Potassium is essential for stomatal regulation, enzyme activation, and ATP synthesis, which are critical for achieving optimal photosynthetic performance [55, 56]. Recent studies indicate that the application of potassium to foliage enhances RuBisCO activity and the electron transport rate within thylakoid membranes, resulting in improved carbon assimilation [57]. Furthermore, PoB has demonstrated the capability to enhance chlorophyll content and stability by mitigating chlorophyll degradation in stress conditions [58], which is consistent with the increased total chlorophyll levels recorded in our study.

Similarly, the notable enhancement in NAR observed under optimal treatments indicates an increased ability of the plants to transform intercepted radiation into biomass. The observed enhancement is due to increased photosynthetic rates and a more efficient conversion of photosynthates into structural biomass [58]. The SEM analysis indicates a strong positive correlation (0.589) between NAR and grain yield, highlighting the significance of this physiological parameter in influencing final yield outcomes. It was recently reported that treatments enhancing NAR had proportionate effects on maize grain yield, highlighting the critical role of photosynthetic efficiency in yield formation [59]. Furthermore, the beneficial effect of photosynthetic parameters, such as TCh and PhR, on GY underscores the essential function of photosynthetic efficiency in yield determination [60].

The substantial rise in grain output under ideal conditions may be ascribed to increased assimilate production, superior allocation to reproductive structures, and effective grain filling. The heightened CGR and enhanced biomass accumulation noted in our study suggest augmented assimilate production, which underpins greater yield potential. The SEM analysis indicated that biomass yield exerted the most significant direct positive effect on grain yield (0.603), implying that both assimilate production and its efficient use and allocation to reproductive organs are vital factors in determining ultimate output. The interplay between source (photosynthetic tissues) and sink (developing grains) strength is crucial in influencing grain filling efficiency and ultimate production [61]. Our findings indicate that the best treatment combination augmented both source activity, via greater photosynthetic efficiency, and sink capacity, as demonstrated by the increased number of grains per line. The balanced improvement of source-sink dynamics enhances assimilate translocation and grain filling, leading to increased grain production [62]. The foliar spray of potassium bicarbonate likely enhanced assimilate transport and grain filling by influencing phloem loading and unloading mechanisms. Potassium is an essential ingredient in phloem transport, enabling sucrose loading into sieve tubes and its subsequent transfer to growing grains. Furthermore, potassium augments the activity of enzymes implicated in starch synthesis within growing kernels, facilitating the effective conversion of transported sucrose into starch [63]. The physiological impacts elucidate the augmented grain filling noted with PoB administration, which facilitated the enhanced harvest index and grain yield.

More importantly, the decrease in oxidative stress indicators (H₂O₂, O₂⁻, and MDA) under ideal conditions signifies improved cellular integrity and metabolic efficacy. Oxidative stress compromises photosynthetic systems, undermines membrane integrity, and reallocates energy resources to defense mechanisms instead of growth and reproduction [64]. The preventive effect of PoB against oxidative damage may be ascribed to its function in activating antioxidant defense mechanisms and stabilizing cellular membranes [65]. The inverse correlation between MDA levels and grain production, as revealed by SEM analysis, underscores the adverse effect of oxidative damage on agricultural output. By alleviating oxidative stress, the ideal treatment combination safeguarded the integrity of the photosynthetic apparatus and upheld effective metabolic activities over the growth season, hence facilitating prolonged assimilate synthesis and translocation [47, 66].

The research revealed notable differences in WUE among various irrigation methods, with drip irrigation exhibiting enhanced performance relative to alternative techniques. The increased WUE is due to the precise water delivery mechanism of drip irrigation, which reduces evaporation losses and ensures optimal distribution within the root zone. The moderate performance of sprinkler irrigation indicates a balanced yet less efficient water distribution pattern, whereas the lower efficiency of surface irrigation arises from heightened water runoff and uneven distribution patterns. The improvement in WUE under mulched conditions illustrates the advantages of this practice, such as decreased soil evaporation, better soil structure, increased water retention, and altered soil temperature patterns [14]. The consistency of these enhancements throughout both growing seasons highlights the effectiveness of mulching as a water conservation method in agricultural systems. Also, the influence of foliar applications on WUE exhibited notable trends, with the PoB treatment showing the most substantial enhancements. These improvements may be linked to PoB’s capacity to boost photosynthetic efficiency, optimize stomatal regulation, and fortify the antioxidant systems of plants. Prior studies have reported analogous results concerning the efficacy of foliar applications in enhancing plant water relations and stress tolerance [67].

In summary, the results indicate that the combination of drip irrigation, rice straw mulching, and potassium bicarbonate foliar application enhances various physiological processes in maize, such as photosynthesis, water relations, assimilate partitioning, and stress tolerance. The physiological improvements outlined contribute to enhanced growth, development, and yield formation in newly reclaimed sandy soils. This provides valuable insights for the sustainable intensification of maize production in resource-limited environments.

## Conclusion

This study demonstrates that the combined use of drip irrigation, rice straw mulching, and potassium bicarbonate foliar application significantly enhances the productivity of *Zea mays* in newly reclaimed sandy soils. The optimal combination of treatments resulted in significant improvements in grain yield (90.1-128.3%) and WUE (90-127.8%) during both experimental seasons compared to conventional agronomic practices. The SEM analysis indicated that biomass accumulation (standardized path coefficient = 0.603), vertical growth metrics (0.594), and photosynthetic efficiency, as assessed by net assimilation rate (0.589), were the most significant positive determinants of grain yield production. In contrast, lipid peroxidation, measured through malondialdehyde activity (-0.434), demonstrated the most considerable negative impact. The integrated management approach resulted in statistically significant improvements in photosynthetic capacity (153.4%) and foliar chlorophyll concentration (54.9%), while also reducing oxidative stress biomarkers, thus clarifying the mechanistic basis for the observed yield increases. Seasonal adaptive physiological responses were observed, marked by a reduction in malondialdehyde accumulation (-34.3%) during the second cultivation cycle, which led to improved productivity parameters despite increased levels of other oxidative stress indicators.

This research enhances the existing agronomic knowledge by offering a detailed quantitative evaluation of the combined effects of hydrological management strategies, soil conservation techniques, and foliar nutritional interventions on *Zea mays* productivity in water-limited sandy soil conditions. The study identifies distinct physiological mechanisms that enhance yield through comprehensive biochemical analyses and quantifies the relative contributions of individual factors to WUE and abiotic stress mitigation using sophisticated statistical modeling techniques.

### Limitations of the study and future prospects

It is imperative to acknowledge the numerous constraints of this investigation. Initially, the results are based on data that was collected over two seasons in a specific geographic area, which may limit their applicability to regions with varying environmental and soil conditions. Secondly, the research was focused on a single maize hybrid, and the efficacy of the interventions that were tested may vary for other varieties. Future research should resolve these limitations by extending the study to multiple locations, diverse maize genotypes, and extended timeframes to evaluate the broader applicability and sustainability of the recommended practices. It will be imperative to examine the economic viability and scalability of trickle irrigation, mulching, and PoB foliar application to encourage their adoption among subsistence farmers. Furthermore, a more comprehensive comprehension of the sustainability of these practices will be achieved by investigating the long-term effects of these practices on soil health, nutrient cycling, and microbial communities. The integration of sophisticated technologies, including precision agriculture and remote sensing, could further optimize resource utilization and improve the efficacy of these management strategies. Lastly, to create comprehensive solutions for enhancing maize productivity in marginal soils, future research should evaluate the potential synergies between these practices and other sustainable agricultural methods, including crop rotation, intercropping, and organic amendments.

## Electronic supplementary material

Below is the link to the electronic supplementary material.

Supplementary Material 1

## Data Availability

All the data are available in the manuscript and with Correspondence authors.

## Abbreviations

PoB: Potassium Bicarbonate
PCA: Principal Component Analysis
SEM: Structural Equation Modeling
PH: Plant Height
NAR: Net Assimilation Rate
CGR: Crop Growth Rate
LAI: Leaf Area Index
NCM: Number of Cobs per Square Meter
NGL: Number of Grains per Line of Cob
HGW: Hundred-Grain Weight
GY: Grain Yield
BY: Biological Yield
LWC: Leaf Water Content
TCh: Total Chlorophyll
PhR: Photosynthetic Rate
TR: Transpiration Rate
H₂O₂: Hydrogen Peroxide
O2˙‾: Superoxide Anion
MDA: Malondialdehyde
WUE: Water use efficiency
