# Supplementary material for: Improving maize yield in newly reclaimed soils: effects of irrigation, mulching, and foliar treatments
Source: BMC Plant Biol. 2025 May 14;25:634. doi: 10.1186/s12870-025-06637-0 (PMC12076918; doi:10.1186/s12870-025-06637-0)
Supplement: Supplementary file 1 — Supplementary Material 1 [file 12870_2025_6637_MOESM1_ESM.pdf]

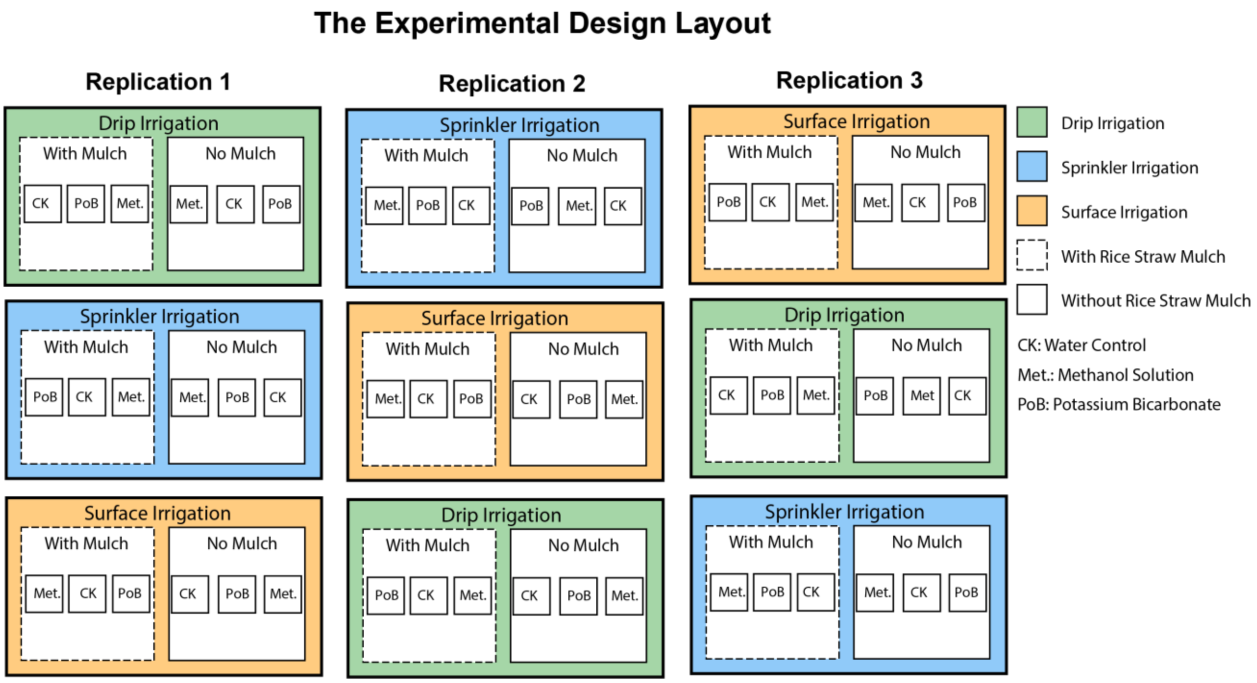

Supplementary Fig. 1. Layout of the split-split plot experimental design with irrigation techniques as main plots, mulching treatments as sub-plots, and foliar spray applications as sub-sub plots, arranged in three replications.
